# Revisiting influenza-hospitalisation estimates from the Burden of Influenza and Respiratory Syncytial Virus Disease (BIRD) project using different extrapolation methods

**DOI:** 10.7189/jogh.14.03017

**Published:** 2024-04-05

**Authors:** John Paget, Sandra S Chaves, You Li, Harish Nair, Peter Spreeuwenberg

**Affiliations:** 1 Nivel, Utrecht, the Netherlands; 2 Foundation for Influenza Epidemiology, Fondation de France, Paris, France; 3 University of Edinburgh, Edinburgh, UK; 4 Nanjing Medical University, Nanjing, China; 5 School of Public Health, University of the Witwatersrand, Johannesburg, South Africa

The World Health Organization (WHO) has estimated that globally, seasonal influenza epidemics result in three to five million cases of severe illness (hospitalisations) every year [1,2]. In a recent paper in the Journal of Global Health, the Burden of Influenza and Respiratory Syncytial Virus Disease (BIRD) project aimed to improve the understanding of influenza-associated hospitalisation estimates at national and global levels [3]. We explored the heterogeneity of the country estimates obtained from the literature by taking into account the role of five study design factors – calculation method, outcome measure, presence of laboratory confirmation, national or sub-national data, and use of single or multi-year data. When we applied this overall estimated pooled rate (40.5 per 100 000 population; 95% confidence interval (CI) = 24.3–67.4) to the world population, we estimated there were, on average, 3.2 million (95% CI = 1.9–5.3) influenza-associated hospitalisations per year [3].

Other research groups have also developed annual global hospitalisation estimates, and these vary widely from 3.2 million based on the BIRD project [3] to 9.46 million (95% CI = 3.7–22.9) estimated from the Global Burden of Disease Study 2017 [4]. There is some difficulty in comparing these estimates (e.g. not all metrics are available), but we found the BIRD estimates were more conservative than those developed by other research groups and are consistent with the WHO estimates of three to five million annual cases [1] and the Centres for Disease Control and Prevention, Influenza Burden Global Project (IceBERG) project (3.95–8.72 million cases per year) [5].

In collaboration with the University of Hong Kong, WHO is currently updating the overall global hospitalisation estimate to make an ‘ensemble’ estimate for seasonal influenza; this is an exercise that is similar to the one done previously by WHO for influenza mortality [6]. As part of this project, we used the 40 national/administrative region estimates that were included in the BIRD project [3] and calculated national, regional and global estimates using two different approaches which incorporate extrapolation procedures that have been used previously (the multiple imputation method and the matching method [7,8]). We present the regional and global BIRD estimates after applying these two methods.

The extrapolation methods have been described elsewhere and used to make global, regional and national burden of disease estimates for influenza [7,8] and respiratory syncytial virus disease (RSV) [9,10]. The indicators used in this model were developed for influenza mortality (e.g. world region, physician density, income levels, population age structure and latitude) [7,8] to make the procedures used for different influenza estimates more homogeneous. We present results for two methods to compare and better validate the different extrapolation methods; the percentages are based on the imputation method, as this method was selected for the final 2009 pandemic influenza mortality estimates (**Table 1**) [7].

**Table 1:** Influenza-associated hospitalisation extrapolations based on the adjusted BIRD estimates (40 countries)*

	All ages					Children (0–4 y)				Elderly (65+ years)			
**Region**	**Number of Stage 1 countries**	Hospitalisation rate (95% CI)*	**Total, n (95% CI)**	**Hospitalisations (%)†**	**Number of Stage 1 countries**		Hospitalisation rate, (95% CI)*	**Total, n (95% CI)**	**Hospitalisations (%)†**	**Number of Stage 1 countries**	Hospitalisation rate, (95% CI)*	**Total, n (%)**	**Hospitalisations (%)†**
World													
*Multiple imputation*	32	66.0 (62.9–69.0)	4 041 600 (3 852 300–4 230 900)	100		28	229.3 (218.2–240.4)	1 458 200 (1 387 600–1 528 800)	36.9	29	152.2 (140.8–163.7)	770 450 (712 420–828 480)	19.5
*Matching*		64.5 (62.6–66.5)	3 955 000 (3 833 400–4 076 500)				205.9 (199.9–211.9)	1 309 300 (1 271 400–1 347 100)			128.8 (121.9–135.6)	651 740 (617 040–686 450)	
WHO AFRO													
*Multiple imputation*	8	60.3 (54.9–65.7)	406 960 (370 550–443 360)	9.4		6	200.6 (181.2–220.0)	271 940 (245 650–298 230)	66.8	7	131.6 (111.6–151.6)	33 705 (28 575–38 834)	8.3
*Matching*		58.9 (55.8–62.1)	397 510 (376 230–418 800)				180.6 (171.1–190.1)	244 870 (231 970–257 770)			104.5 (93.2–116.0)	26 784 (23 858–29 710)	
WHO EMRO													
*Multiple imputation*	1	53.2 (45.3–61.1)	259 370 (220 840–297 890)	6.0		1	198.9 (170.5–227.3)	137 080 (117 520–156 650)	52.9	2	133.9 (104.6–163.3)	31 107 (24 299–37 915)	12.0
*Matching*		60.3 (55.3–65.3)	294 060 (269 580–318 540)				208.2 (193.2–223.2)	143 470 (133 130–153 820)			121.2 (104.1–138.4)	28 150 (24 165–32134)	
WHO EURO													
*Multiple imputation*	7	44.3 (39.2–49.4)	369 650 (326 920–412 390)	8.5		3	228.5 (210.1–246.9)	120 200 (110 520–129 870)	32.5	5	118.2 (99.2–137.2)	152 170 (127 700–176 640)	41.2
*Matching*		52.6 (49.5–55.6)	439 000 (413 520–464 490)				181.5 (171.9–191.0)	95 455 (90 436–100 470)			115.1 (104.2–126.1)	148 270 (134 120–162 410)	
WHO PAHO													
*Multiple imputation*	6	69.2 (62.8–75.6)	581 950 (528 140–635 760)	13.4		8	191.6 (168.6–214.6)	147 440 (129 750–165 140)	25.3	9	194.4 (170.6–218.1)	156 010 (136 950–175 070)	26.8
*Matching*		66.5 (62.7–70.3)	559 090 (527 390-590 790)				193.1 (182.1–204.0)	148 550 (140 150-156 940)			153.9 (140.6–167.3)	123 530 (112 810–134 240)	
WHO SEARO													
*Multiple imputation*	3	105.5 (94.6–116.4)	1 673 500 (1 500 400–1 846 600)	38.6		3	250.7 (211.5–290.0)	463 590 (391 060–536 120)	27.7	1	193.4 (152.9–233.9)	175 930 (139 090–212 770)	10.5
*Matching*		83.8 (77.2–90.5)	1 329 500 (1 223 700–1 435 300)				218.3 (198.8–237.9)	403 680 (367 480–439 870)			139.1 (115.1–163.0)	126 480 (104 670–148 290)	
WHO WPRO													
*Multiple imputation*	7	63.2 (55.1–71.3)	1 046 000 (911 980–1 180 000)	24.1		7	305.5 (276.5–334.6)	344 880 (312 040–377 720)	33.0	5	141.8 (111.8–171.8)	216 600 (170 720–262 480)	20.7
*Matching*		65.1 (60.3–69.9)	1 077 600 (998 500–1 156 700)				253.7 (239.6–267.9)	286 410 (270 460–302 360)			138.7 (121.3–156.2)	211 880 (185 210–238 540)	

AFRO – African Region, EMRO – Eastern Mediterranean Region, EURO – European Region, PAHO – Pan American Region, SEARO – South-East Asian Region, WHO – World Health Organization, WPRO – Western Pacific Region

*All rates are per 100 000 population. The percentages are based on the Multiple Imputation extrapolations only. The number of hospitalisations is based on the world population in 2008.

†The percentage of the total distribution of hospitalisations by region and age group is based on the Multiple Imputation Method.

For the data inputs, we had 32 country-level influenza-associated hospitalisation estimates for the all-age extrapolations, 28 for children aged 0–4 years, and 29 for the elderly (65+ years) (**Table 1**). Importantly, we had at least one country-level estimate for all three age groups in all eight WHO regions. The total number of country estimates and the regional distribution of the estimates are comparable to previous global extrapolation exercises (e.g. we only had 20 estimates for the 2009 pandemic influenza mortality estimate [7], and we did not have estimates for one WHO region for the seasonal influenza mortality estimates [8]).

Importantly, the overall estimate has increased from 3.2 million (95% CI = 1.9–5.3) [3] to 3.96 million (95% CI = 3.83–4.08) for the matching method and to 4.04 million (95% CI = 3.85–4.23) for the multiple imputation method. The main reason for the increase is that the original BIRD estimate applied the pooled global rate to the world population [3], whilst the new estimates calculate the number and rate (with 95% CIs) for each country, region and the world using a model. The model uses a distribution of plausible rates for each country as input [7], with the rates being based on the original 40 meta-analysis adjusted country estimates from the BIRD study [3] and the relation with the indicator set using the previously mentioned methods [7,8]. The new estimates are more refined and better capture country and regional differences in hospitalisation rates and the population structures of each country.

Our estimates vary by extrapolation method, with the matching method providing slightly more conservative estimates. This is an outcome that was previously seen with the 2009 pandemic mortality estimates and is linked to the multiple imputation method, allowing greater variability in the country-specific estimates [7]. The estimates also vary by reference population denominator, based on a reference world population of 2008 (which represents the year when most estimates used to make the BIRD estimates were available [3]; range 1979–2019), i.e. a world population of 6.8 billion. Importantly, if we used a reference population denominator of 2019, i.e. a world population of 7.7 billion, one would have 5.09 million influenza-associated hospitalisations for all ages (imputation method) (**Table 1**).

In terms of rates per 100 000, the highest all-age rates (multiple imputation and matching methods) were estimated in the Southeast Asia region (which includes India) and the lowest in the Europe region. For the total number of hospitalisations, the Southeast Asia region (39% of global influenza-associated hospitalisations) and the Western Pacific region (which includes China, 24%) had the highest numbers. These two regions (which represent 53% of the world population) had 63% of the total hospitalisations.

For children aged 0–4 years, the highest rates per 100 000 population (using multiple imputation and matching methods) were estimated in the Western Pacific region and the lowest in the American (multiple imputation) and sub-Saharan Africa (matching) regions. Regarding the percentage of total hospitalisations in the zero to four years age group, the sub-Saharan Africa region had the highest percentage contribution (67%), and the American region had the lowest (25%).

For the elderly (65+ age group), the highest rates per 100 000 population (multiple imputation and matching methods) were estimated in the American and South-East Asian region, and the lowest in the European (multiple imputation) and sub-Saharan Africa regions (matching). Nonetheless, regarding the percentage of total cases in the 65+ age group, the European region stood out with 41% of all influenza hospitalisations, and the lowest percentage was in the sub-Saharan Africa region with 8% of all cases.

As part of a sensitivity analysis, we assessed the impact of not using the pooled country estimates, which take into account the five different factors [7], as inputs for the extrapolations. If we use the raw estimates extracted from the English and Chinese-language literature reviews [7] as inputs for the extrapolations, the estimated influenza-associated hospitalisations for all ages (imputation method) rose substantially from 4.04 to 8.5 million (2008 population reference).

In summary, our new estimates for 2008–19 align well with the WHO estimates [1] but are on the lower side compared to other research groups [3]. This confirms and underlines the difficulty of making influenza-associated hospitalisation estimates, reflected in the heterogeneity of estimates within and across countries [3,11]. Our study also highlights the sensitivity of the global estimates to the population denominator (e.g. 2008 or 2019) and whether the country estimates entered into the extrapolation procedure are adjusted [7] or not (using the raw hospitalisation rates more than doubles our global estimate). The latter has important methodological implications for other research groups using similar data inputs to estimate their global and regional influenza-associated hospitalisations.

From a public health perspective, our study highlights the high burden of influenza-associated hospitalisations in Asia (the WHO South-East Asian and Western Pacific regions) and a differing age signature by world region (the percentage of cases aged from zero to four years old and 65+ years in each region). The latter probably reflects different population structures (e.g. the high percentage of hospitalisations in the elderly in Europe) and differing hospitalisation practices around the world (e.g. the lower health-seeking behaviour among older persons in sub-Saharan Africa region [12]). These findings strengthen the need to assess and analyse the burden of influenza-associated hospitalisations at a regional (and country) level.
